# A Cross-Sectional Study of the Prevalence of Metabolic Syndrome and Associated Factors in Colombian Collegiate Students: The FUPRECOL-Adults Study

**DOI:** 10.3390/ijerph14030233

**Published:** 2017-02-27

**Authors:** Javier Martínez-Torres, Jorge Enrique Correa-Bautista, Katherine González-Ruíz, Andrés Vivas, Héctor Reynaldo Triana-Reina, Daniel Humberto Prieto-Benavidez, Hugo Alejandro Carrillo, Jeison Alexander Ramos-Sepúlveda, Emilio Villa-González, Antonio García-Hermoso, Robinson Ramírez-Vélez

**Affiliations:** 1 Grupo GICAEDS, Facultad de Cultura Física, Deporte y Recreación, Universidad Santo Tomás, Bogotá DC 110311, Colombia; javiermartinezt@usantotomas.edu.co; 2 Centro de Estudios para la Medición de la Actividad Física «CEMA», Escuela de Medicina y Ciencias de la Salud, Universidad del Rosario, Bogotá DC 111221, Colombia; jorge.correa@urosario.edu.co (J.E.C.-B.), danielprietob@gmail.com (D.H.P.-B.); 3 Grupo de Ejercicio Físico y Deportes, Vicerrectoría de Investigaciones, Universidad Manuela Beltrán, Bogotá DC 110231, Colombia; katherine.gonzalez@docentes.umb.edu.co (K.G.-R.); jose.vivas@docentes.umb.edu.co (A.V.); 4 Grupo GRINDER, Programa de Educación Física y Deportes, Universidad del Valle, Santiago de Cali DC 760010, Colombia; hectortriana@usantotomas.edu.co (H.R.T.-R.); hugo.carrillo@correounivalle.edu.co (H.A.C.); 5 Facultad de Educación a Distancia y Virtual, Institución Universitaria Antonio José Camacho, Santiago de Cali DC 760010, Colombia; jeisonandres@yahoo.es; 6 Department of Education Sciences, University of Almería, Almería DC 04120, Spain; evilla@unach.edu.ec; 7 PROFITH “PROmoting FITness and Health through Physical Activity” Research Group, Department of Physical Education and Sport, School of Sport Sciences, University of Granada, Granada DC 18010, Spain; 8 Laboratorio de Ciencias de la Actividad Física, el Deporte y la Salud, Facultad de Ciencias Médicas, Universidad de Santiago de Chile, USACH, Santiago DC 9160030, Chile; antonio.garcia.h@usach.cl

**Keywords:** prevalence, cardiometabolic risk factors, obesity, cardiovascular disease

## Abstract

Metabolic syndrome (MetS) is one of the major public health problems worldwide. The objective of the present study is to investigate the prevalence and the associated variables of MetS in Colombian collegiate students. This cross-sectional study included a total of 890 (52% women) healthy collegiate students (21.3 ± 3.2 years old). The prevalence of MetS was determined by the definition provided by the International Diabetes Federation (IDF). We further examined associations between the prevalence of MetS and related factors, such as age, gender, anthropometric and body composition, weight status, and nutrition profile. The overall prevalence of MetS was 6.0% (95% CI = 4.5% to 7.6%), and it was higher in men than women. The most prevalent components were low high-density lipoprotein cholesterol, high triglyceride levels, waist circumference, and blood pressure levels. The predisposing factors for having a MetS included: being male, over 23 years old, overweight or obese, and having an unhealthy waist-to-height ratio. In conclusion, the occurrence of MetS in young adults is substantial. These findings may be relevant to health promotion efforts for collegiate students in order to develop prospective studies and screening for young adults, which will aid in targeted intervention development to decrease cardiometabolic risk factors.

## 1. Introduction

Metabolic syndrome (MetS) is a major public health problem worldwide [1]. A diagnosis of MetS is based on the existence of pre-diabetes combined with dyslipidemia (elevated levels of total or low-density lipoprotein cholesterol, or low high-density lipoprotein cholesterol levels), elevated blood pressure and central adiposity [2,3]. The screening and early diagnosis of MetS are, however, not easy in young subjects, because the diagnostic criteria for the estimation of MetS has not been fully established [3]. Identifying individuals with MetS is important due to its association with an increased risk of coronary heart disease and Type 2 diabetes mellitus [4,5,6,7,8]. For this reason cardiovascular risk factor measurements are important, even at early age, to detect a risk profile in time for intervention.

On the other hand, several risk factors for MetS have been suggested, such as weight status [9], measures of central adiposity and inflammatory markers [10], dietary factors, such as the intake of total fat or saturated fat [11], physical inactivity [12], and poor physical fitness [13]. We previously demonstrated in children and adolescents aged 9–17 years that those with the highest values of body mass index and subjects aged 9–12 years old were more likely to have a prevalence of MetS [3]. Additionally, other studies that included diverse Hispanic/Latino populations suggested a marked heterogeneity in risk factor prevalence within this population [7,8].

Currently, there is no gold standard diagnostic criteria for MetS in adults, and a description of the prevalence of MetS according to the few proposed definitions is necessary for different populations around the world [4,5]. For example, the National Health and Nutrition Examination Survey reported that when using the National Cholesterol Education Program definition of the MetS [4,6], the age-adjusted prevalence of the MetS was 44.5% among Hispanic men, and 44.1% among Hispanic women [7,14,15,16]. Furthermore, previous studies in the Colombian population have shown an inconsistent association between the component cut-offs specified in diagnostic criteria for MetS and its utility [17,18].

The lifestyle of the college population has changed considerably over the past 20 years due to a rapid improvement in socioeconomic status [19,20]. These changes, in addition to the adoption of a western lifestyle and diet, have led to a rise in the prevalence of overweight and obesity in Colombians, particularly among university students [17]. Other studies has reported MetS in younger populations but use a much wider age range [3] or include non–college students [7,8,9]. Additionally, it was challenging to clearly define the “young adult” age group. In previously reported cross-sectional studies, the MetS prevalence in adolescents included ages 12–18 years [3] or 10–19 years old [16]. Having one international definition for the “young adult” age group would be helpful for future data comparisons.

Based on the International Diabetes Federation Task Force on Epidemiology and Prevention, National Heart, Lung, and Blood Institute, American Heart Association, World Heart Federation, International Atherosclerosis Society, and the International Association for the Study of Obesity (IDF/NHLBI/AHA/WHF/IAS/IASO-2009) definition of MetS [21], the objective of the present study is to investigate the prevalence and the associated variables of MetS in Colombian collegiate students. In young adults, these factors may also contribute to the identification of the common and/or distinctive features of MetS, which can essentially aid in understanding the background of this disease and its components. Therefore, it is necessary to identify high-risk collegiate students in order to examine the associated factors for MetS.

## 2. Methods

### 2.1. Study Design and Sample Population

We performed cross-sectional analyses of baseline data from participants in the FUPRECOL study (Association between Muscular Strength and Metabolic Risk Factors in Colombia), which focused on the associations between fitness, health, and non-communicable diseases. We have recently published a complete description of the FUPRECOL study design, methods, and primary outcomes for our current cohort [22,23]. A total of 890 volunteers (52.0% female, mean age = 21.4 (SD 3.3) years old) between the ages of 18 and 30 years, of low to middle socioeconomic status (SES: 1–4 on a scale of 1–6 defined by the Colombian government), were enrolled in public or private universities in the capital district of Bogota and Cali, Colombia. Inclusion criteria were: no self-reported history of inflammatory joint disease or neurological disorder; and not an athlete participating at an elite level. Volunteers were not compensated for their participation. Subjects with a medical or clinical diagnosis of a major systemic disease (including malignant conditions such as cancer), type 1 or 2 diabetes, high blood pressure, hypothyroidism/hyperthyroidism, a history of drug or alcohol abuse, regular use of multivitamins, or inflammatory (trauma, contusions) or infectious conditions, and ≥35 kg/m^2^ body mass index (BMI) were also excluded from the study. The Institutional Ethics Committee in accordance with the latest version of the Declaration of Helsinki approved the study (UMB No. 01-1802-2013). After reading and signing an informed consent form in order to participate in the study, volunteers were given an appointment for a testing session at the university laboratories. The students who agreed to participate and who had signed the informed consent form were given appointments for the following procedures. Students were informed that their participation was voluntary with no penalty for not participating.

### 2.2. Data Collection

All data were collected at the same time in the morning, between 7:00 and 10:00 a.m. Body weight and height were measured following standard procedures and using an electronic scale (Tanita^®^ BC544, Tokyo, Japan) and a mechanical stadiometer platform (Seca^®^ 274, Hamburg, Germany), respectively. BMI was calculated as body weight in kilograms divided by the square of the height in meters. BMI was classified as underweight, normal weight, overweight, or obese using the World Health Organization (WHO) criteria [24]. Waist circumference (WC) was measured at the midpoint between the last rib and the iliac crest using a tape measure (Ohaus^®^ 8004-MA, Parsippany, NJ, USA). To classify WC, we used criterion-referenced health-related cut-points derived from a cross-sectional, Colombian national representative nutrition survey (ENSIN, 2010) [25]. In addition, we also calculated the waist-to-height ratio (WHtR). Ramírez-Vélez et al. [25] proposed a universal WHtR cut-off of ≥0.50, which might identify early cardiovascular risk in Colombian adults. Lean mass (kg) and body fat percentage (BF%) were determined for bioelectrical impedance analysis (BIA) by Tanita BC-418^®^ (Tokyo, Japan). Measurements were made with the participant in a standing position with arms and legs lying parallel to the trunk and separated, so that the thighs were not touching. Before testing, participants were required to adhere to these BIA instructions: (i) to not eat or drink within 4 h of the test; (ii) to not consume caffeine or alcohol within 12 h of the test; (iii) to not take diuretics within seven days of the test; (iv) to not do physical exercise within 12 h of the test, and; (v) to urinate within 30 min of the test [26]. An electrical current of 50 kHz was passed through the participant and the resistance and reactance were measured. To ensure data quality, the equipment was calibrated daily using a known calibration standard, in accordance with Ramírez-Velez et al.’s standard procedures [22]. The in vivo coefficient of variation assessed in our laboratory for adults use was 1.2% for BF% [22]. The BF% cut-offs chosen to perform the sensitivity and specificity comparisons were the values most frequently cited by international scientific literature (20%–30%; 30%–40%, and ≥40% adiposity) by bioelectrical impedance analysis (BIA) in the Spanish population [27].

Blood pressure was measured twice from the left hand, via an Omron M6 Comfort (Omron^®^ Healthcare Europe B.V., Hoofddorp, The Netherlands) while the participants were sitting still. The blood pressure monitor cuff was placed two to three finger-widths above the bend of the arm and a two-minute pause was allowed between the first and the second measurements. The mean blood pressure (MBP) was calculated using the following formula: MBP = (systolic blood pressure + (2 × diastolic blood pressure))/3.

Food consumption was assessed using the Kidmed questionnaire [28]. This tool consists of sixteen questions related to the principles of Mediterranean dietary patterns as reported previously [28]. In this study, we divided participants into two groups: less or equal to 8 points (ideal healthy diet), and less or equal to 7 points (non-ideal healthy diet).

### 2.3. Laboratory Measurements

After fasting for 12 h, blood samples were obtained from a capillary sample at 6:30 am–7: 00 am. Participants were asked to not participate in any prolonged exercise for the 24 h prior to testing. The biochemical profile included: (1) the plasma lipid triglycerides, total cholesterol, high-density lipoprotein cholesterol (HDL-c), fasting glucose, and low-density lipoprotein cholesterol (LDL-c) (by enzymatic colorimetric methods). Inter-assay reproducibility (coefficient of variation) was determined from 80 replicate analyses of eight plasma pools over 15 days, and shown to be 2.6%, 2.0%, 3.2%, and 3.6% for triglycerides, total cholesterol, HDL-c, and LDL-c, respectively, and 1.5% for serum fasting glucose.

### 2.4. Covariates

A standardized questionnaire, (family, activity, nutrition, tobacco toxins, alcohol, sleep/stress, personality type, insight, career) FANTASTIC lifestyle, was used to collect comprehensive information about substance use via a personal interview with participants [23]. Alcohol drinkers and tobacco smokers were defined, respectively, as subjects who had consumed any alcoholic beverage ≥1 times per week, and those who had smoked ≥10 cigarettes per week, for at least six months. The determination of the physical activity (PA) levels was measured using the FANTASTIC questionnaire [23]. Although the FANTASTIC questionnaire only refers to PA participation in the previous week, subjects were also asked whether the pattern of PA reported in the FANTASTIC questionnaire was consistent with the previous seven days. PA was categorized as follows: insufficient: no PA practice (<150 min/week), OR sufficient PA: five or more days of moderate-intensity PA and/or walking, in combination or alone, at least 30 min/day, accumulating a minimum of 150 min/week according to WHO recommendations. The accuracy of information about substance use and PA levels obtained from questionnaires has been validated by different experiments and is described in detail elsewhere [23].

### 2.5. Definition of the MetS

MetS was defined as including ≥3 of the following metabolic abnormalities [29,30,31,32,33,34,35,36,37]: WC ≥90 cm in men or ≥80 cm in women; HDL-c <40 mg/dL in men or <50 mg/dL in women; triglyceride ≥150 mg/dL; fasting glucose ≥100 mg/dL; systolic BP (SBP) ≥130 mmHg; and/or diastolic BP (DBP) ≥85 mmHg.

### 2.6. Ethics Statement

The sample size was calculated using Minitab software (version 16, Minitab Inc., State College, PA, USA) and was based on an expected prevalence of 4% MetS. At 0.80 power and a 0.05 significance level, a sample size of 600 would achieve a 1.60% margin of error for the survey of the college student population. Data analyses were carried out using IBM SPSS 21 (SPSS, Inc., Chicago, IL, USA). Descriptive statistics were computed and summarized; continuous variables were summarized using means and standard deviations (SD), and categorical variables using proportions (%). The normality of the selected variables was verified using histograms and Q-Q plots. Differences were analysed using a *t*-student or chi-square test (χ^2^) in order to explore sex-group differences. Binary logistic regression analysis was used to study the association between anthropometric (sex and age), body composition (levels of adiposity and WHtR), nutritional status (BMI categories), and nutrition profile (Mediterranean diet quality (low, medium, and high)), and the presence of MetS as the outcome variable, adjusted by smoking, alcohol intake, PA levels, and BF%. Odds ratios were considered a confounder if they shifted the model in a constant direction with a proportional increase in the exposure level with a *p* value < 0.10. The level of statistical significance was established at *p* < 0.05.

## 3. Results

### 3.1. Descriptive Characteristics

Table 1 shows the demographic descriptive statistics of the sample. The final sample had a mean age of 21.3 ± 3.2 (range 18.0–30.0) years and contained slightly more females (52%). Women had lower levels of weight, height, WC, WHtR, lean mass, blood pressure, and triglycerides than men (*p* < 0.05). In women, the prevalence of overweight and obesity were 20.3% and 5.6%, and these were 24.1% and 5.4% in men, respectively (*p* < 0.05), according to the WHO criteria, but women also showed a higher Mediterranean diet adherence (i.e., in level 2 and 3) compared to men (*p* < 0.001). The overall prevalence of MetS was 6.0% (95% CI = 4.5% to 7.6%), higher in men than women. Men were higher in three or more components of the MetS criteria than women (9.0% vs. 3.0%, *p* < 0.05). At least one MetS component was found in 331 participants (37.2%); two MetS components were present in 151 participants (17.0%); three MetS components were found in 39 participants (4.4%); and four or more components of MetS were present in 14 participants (1.6%) (see Table 1).

### 3.2. Clinical Characteristics and Distribution by Metabolic Syndrome Status

Participants with MetS had a significantly higher weight, body mass index, WC, waist to height ratio, body fat, blood pressure, and serum metabolic biomarkers (*p* < 0.001) (see Table 2). Furthermore, the most prevalent MetS variables in the sample were within men; over 23 years, with levels of adiposity of 20%–29%, overweight status, a medium Mediterranean diet adherence, and Afro-Colombian ethnicity showed a higher MetS (73.6%, 49.1%, 50.9%, 58.5%, 64.2%, and 67.9%, respectively).

### 3.3. Factors Associated with Metabolic Syndrome

**Figure 1:**
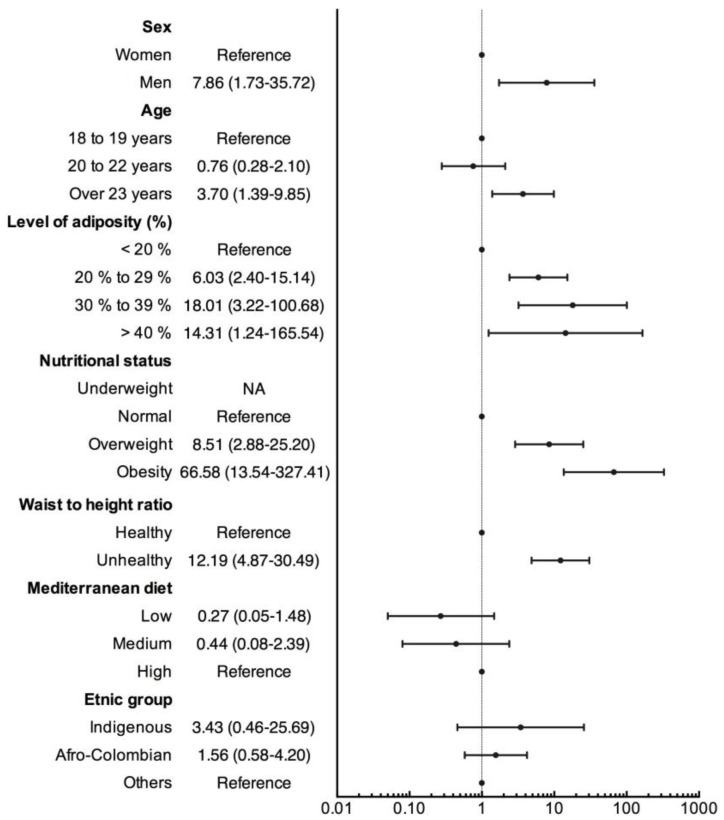
Factors associated with MetS among a sample of college students from Colombia.

Figure 1 shows the results from the logistic regression analysis. The predisposing factors for having a MetS included being male (OR 7.86 (95% CI = 1.73 to 35.72)), being over 23 years old (OR 3.70 (95% CI = 1.39 to 9.85)), having 20%–29% adiposity (OR 6.03 (95% CI = 2.40 to 15.14)), being overweight (OR 8.51 (95% CI = 2.88 to 25.20)), being obese (OR 66.58 (95% CI = 13.54 to 327.41)), and having an unhealthy waist-to-height ratio (OR 12.19 (95% CI = 4.87 to 30.49)).

## 4. Discussion

The main finding of the present study was a MetS prevalence of 6%, which is an intermediate value compared to those reported in local and international studies, ranging from 2% to 13% (29–38). For example, Dalleck et al. [34] reported a prevalence of 6.8% in university students with similar characteristics to those found in the present study. Ford et al. [35] conducted a cross-sectional study with adults aged 18–30 years in the United States and reported a similar MetS prevalence of 6.7%, using the definition of the National Cholesterol Education Program–Adult Treatment Panel III of the United States.

The rates found in university contexts by the present study were much higher than those found by the following authors: Huang et al. [29] in 163 students aged 18–24 years in Kansas, United States, (0.6%); de Freitas et al. [30] in 702 Brazilian university students (1.7%); Fernandes et al. [31] in 189 students aged 18–24 years (3.7%); Yen et al. [32] in 8226 students with a mean age of 19.2 ± 2.3 years (4.6%); and Burke et al. [33] in 1701 students aged 18–24 years, who were enrolled in an introductory nutrition course and had met the age requirements of the University of New Hampshire’s Young Adult Health Risk Screening Initiative (4.9%). On the other hand, our rates were lower than those found by Ruano et al. [36] in 796 Spanish students aged 17–25 years (7.5%), and Mattsson et al. [38] in 2182 healthy young adults (1007 men and 175 women) aged 24–39 years (13%). Clearly, the prevalence of MetS could differ between studies depending on the MetS cluster used, design method, and target population. In this study, we have used the International Diabetes Federation (IDF) and American Heart Association (AHA) and the National Heart, Lung, and Blood Institute (NHLBI) joint statement, as it was an international attempt to harmonize the definition of MetS; central obesity is not an obligatory component of this definition and it is ethnic specific.

The criterion for MetS that had the highest prevalence (40.7%) was low HDL-c. Previous studies have described the prevalence of low HDL-c with values between 20.1% and 47.8% [31,34,39]. From a preventive perspective, HDL-c could be an early marker for the development of metabolic disorders [40] and, therefore, strategies such as weight control, caloric intake reduction, and regular physical exercise should be encouraged in university contexts.

High blood pressure is an important risk factor for the main cardiovascular disorders, such as ischemic heart disease and stroke [41]. Several observational studies have described the frequent association between high blood pressure values and MetS, however, the interrelationship with obesity or other risk situations, such as alterations in glucose metabolism, suggests that the basis of this epidemiological association could be related to common physiopathological implications [42,43].

In the present study, we demonstrated that the elevation of blood pressure was the second most prevalent criterion, with a frequency of 20.9%. This result was similar to that reported by the The National Health and Nutrition Examination Survey (NHANES) (2003–2006). This finding is important because most longitudinal studies have agreed that a high blood pressure level is one of the most prevalent criteria [44], and individuals diagnosed with high blood pressure have high MetS prevalence [45].

When we compared sex-related differences, it was noted that men had a MetS prevalence of 9.1%, which was three times higher than the prevalence observed in women (3%). The occurrence of MetS among men was significant (OR = 3.22; 95% CI = 1.76 to 6.03). This result is opposed to that reported by Abda et al. [46], who found that there was greater occurrence of MetS in women (OR = 2.86; 95% CI = 1.51 to 5.42); however, our findings were in line with the data reported by de Freitas et al. [30] in Brazil, who observed that the prevalence of MetS in men was twice as high than in women. Barbieri et al. [47] reported that the prevalence of MetS was 2.20 times higher in men than in women (10.7 vs. 4.2%). On the other hand, Ramírez-Vélez et al. [3] conducted a local study with Colombian youth and observed a slightly higher prevalence in girls than in boys when MetS was estimated using Ferranti’s criteria (12% vs. 9.8%). This same result was reported by Fernandes et al. [31] in the United States population. These authors found higher rates of MetS in women than in men (4.7% vs. 1.6%). Similar results were observed by Park et al. [48] in Korean young adults aged 20–39 years.

With respect to age, we observed a significantly increased prevalence of MetS, ranging from 3.8% in the group of individuals aged 18–19 years to 11% in the group of individuals aged 23 years or older. In fact, it has been reported that the risk of MetS and the related criteria increase with age [49]. In the present study, we observed that university students aged over 23 years had a greater occurrence of MetS (OR = 3.04; 95% CI = 1.50 to 6.16), a result that was in line with rates found in Arab women [50]. These authors suggested that the risk of MetS increases after 23 years of age (OR = 2.96; 95% CI = 1.31 to 6.06). Dhaheri et al. [20] reported a similar tendency in a cohort of the study “Young Female Emirati Adults”, in which the prevalence of MetS ranged from 4.1% among university students aged 17 to 19 years, to 11.3% among students aged 23–25 years.

It has been reported that central obesity is one of the main characteristics of MetS, particularly due to increased values of free fatty acids in the blood and the inhibition of insulin action in peripheral tissues [50,51,52]. In the present study, we observed 10.7% of central obesity in the population assessed, a value that increased to 56.6% in individuals with MetS. This risk factor was also described as the most prevalent metabolic abnormality in the “Young Adult Health in Saudi Arabia” study [53], and by Park et al. [48] in Korean young adults aged 20–39 years.

The risk of MetS in overweight and obese individuals was 49.2 (95% CI = 18.07 to 133.97) and OR = 49.12 (95% CI = 18.07 to 133.97), respectively. This result was in line with the findings of the following authors: Dhaheri et al. [50] in university women from the United Arab Emirates (OR = 11.19; 95% CI = 3.06 to 40.86); Ismail in adult Indians [54] (OR = 7.17; 95% CI = 1.73 to 19.7); and Ramírez-Velez et al. [3] in Colombian children and adolescents (OR = 1.69; 95% CI = 1.13 to 2.53). Similarly, we observed that overweight increased the risk of MetS (SM) (OR = 18.95; 95% CI = 7.78 to 46.19). This result was similar to that reported by Abda et al. [46] in Ethiopian adults using the National Cholesterol Education Program–Adult Treatment Panel III (OR = 5.49; 95% CI = 2.90 to 1054).

Ethnicity has been associated with the development of MetS worldwide [55]. According to the definition of the National Cholesterol Education Program–Adult Treatment Panel III of the United States, the prevalence of MetS in adults was: 32% in Hispanic Americans; 22% in African Americans; and 24% in European Americans [56]. Similarly, a prevalence of 24% in Caucasian individuals was reported in the Framingham Study, and a prevalence of 31% in individuals of Mexican origin was reported in the San Antonio Heart Study [57]. However, South America has no standards for the diagnosis of MetS. In this respect, Ramírez-Vélez et al. [58,59] reported a high prevalence of micronutrient and vitamin deficiencies combined with the double burden of nutritional disorders in indigenous and Afro-Colombian populations, a condition related to metabolic disorders as described in the present study. When we grouped the individuals by ethnicity, the indigenous and Afro-Colombian individuals exhibited a higher prevalence of MetS, with values of 20% and 8%, respectively.

Previous studies have reported that several alterations in body composition and metabolic pathways could be in part explained by genetic and ethnic factors [60]. For example, the HDL-c metabolism has shown the highest heritability estimates (between 50% and 60%), whereas systolic blood pressure showed the lowest heritability estimates (between 6% and 18%). Much of the risk of MetS associated with ethnicity can be explained by the change in steroid hormone levels and the metabolism of carbohydrates and lipids [61]. An increase in total fat and the distribution of central fat, resulting from alterations in hormones, such as leptin, adiponectin, resistin, and estrogen, which are mediators of MetS, has been reported in indigenous and Afro-American populations. The increased susceptibility of Africans when exposed to lifestyle abnormalities, such as high salt intake, shows the collision between genes adapted to a warm climate and the “modern” lifestyle [62].

Studies based on adherence to the Mediterranean diet reported controversial results [63,64,65,66]. The AHA includes diet behavior as one of seven health behaviors to track for the 2020 Strategic Impact Goals [63] and more recently, has been evaluated for the prevention and treatment of MetS [64]. Consistent with our findings, we did not detect a significant effect of healthy diets on the prevalence of MetS [65]. Babio et al. [66] showed a positive association between adherence to the Mediterranean diet and a lower odds ratio of having MetS in a population with a high risk of cardiovascular disease, while Tzima et al. [67] observed a modest, not-significant association between insulin resistance and adherence to the Mediterranean diet in overweight/obese subjects. Overall, the adoption of the Mediterranean diet has been demonstrated to reduce MetS by 66%; moreover, this effect seems to be independent of weight loss [66].

One limitation of the present study was the lack of information about physical fitness levels (e.g., cardiorespiratory fitness or musculoskeletal fitness) and family history of cardiometabolic diseases or non-alcoholic fatty liver disease (NAFLD) detection, which suggests the need for more robust analyses. Another limitation was the cross-sectional design of the study, which did not allow a “cause-effect” relationship to be established. These and other questions deserve further investigation by future well-designed longitudinal studies. On the other hand, the relevance of the present study lies in the fact that it is the first study to assess MetS prevalence in a Colombian university population.

## 5. Conclusions

In summary, we found that the prevalence of MetS and its components was substantial in the studied population. Dyslipidemia, followed by central obesity and high blood pressure levels, were the most frequent components of MetS. Being male, over 23 years old, overweight or obese, and a unhealthy WHtR were significantly associated with MetS. Interventions that take into account nutritional elements and physical activities are recommended, because they can counteract this important health problem in university contexts.

## Figures and Tables

**Table 1:** Characteristics among a sample of college students from Colombia (mean (SD) or [frequencies]).

	All Participants (*n* = 890)	Women (*n* = 463)	Men (*n* = 427)	*p* Value
Age (years)	21.3 (3.2)	21.4 (3.1)	21.3 (3.3)	0.478
Weight (kg)	64.2 (12.5)	58.8 (10.0)	69.8 (12.4)	<0.001
Height (cm)	165.8 (9.0)	159.8 (6.1)	172.4 (6.7)	<0.001
Body mass index (kg/m^2^)	23.2 (3.7)	23.0 (3.7)	23.4 (3.6)	0.097
Nutritional status by BMI [%] *				
Underweight	29 [3.3]	16 [3.5]	13 [3.0]	0.588
Normal weight	615 [69.1]	327 [70.6]	288 [67.4]	
Overweight	197 [22.1]	94 [20.3]	103 [24.1]	
Obese	49 [5.5]	26 [5.6]	23 [5.4]	
Body composition				
Waist circumference (cm)	75.4 (9.6)	72.0 (8.0)	79.0 (9.7)	<0.001
Waist-to-height ratio	0.455 (0.055)	0.452 (0.053)	0.455 (0.055)	0.035
Body fat (%)	21.6 (8.8)	26.8 (7.2)	16.0 (6.6)	<0.001
Lean mass (kg)	49.7 (9.7)	42.6 (4.1)	58.4 (7.2)	<0.001
Level of adiposity [%] *				
<20	413 [46.4]	88 [19.0]	325 [76.1]	<0.001
20 to 29	317 [35.6]	229 [49.5]	88 [20.6]	
30 to 39	140 [15.7]	127 [27.4]	13 [3.0]	
>40	20 [2.2]	19 [4.1]	1 [0.2]	
Mediterranean diet quality [%] *^,a^				
Low/medium/high	[54.8/36.2/8.9]	[42.8/42.8/14.5]	[61.0/32.9/6.1]	<0.001
Ethnic group [%] *				
Indigenous/Afro-Colombian/Mestizo	[2.2/25.3/72.5]	[2.0/32.2/65.8]	[2.4/21.7/75.9]	0.052
Cardiovascular risk factors				
Systolic blood pressure (mmHg)	117.9 (12.6)	112.5 (11.0)	123.7 (11.7)	<0.001
Diastolic blood pressure (mmHg)	74.1 (9.8)	72.0 (9.4)	76.7 (10.8)	<0.001
Mean blood pressure (mmHg)	88.7 (9.7)	85.5 (8.8)	92.4 (9.7)	<0.001
Total cholesterol (mg/dL)	142.2 (33.6)	148.7 (34.4)	135.4 (31.3)	<0.001
Triglycerides (mg/dL)	95.3 (48.9)	92.2 (47.2)	98.9 (51.0)	0.040
LDL-c (mg/dL)	84.0 (27.3)	86.0 (27.8)	81.7 (26.5)	0.028
HDL-c (mg/dL)	44.0 (12.8)	47.8 (13.4)	39.8 (10.7)	<0.001
Glucose fasting (mg/dL)	83.3 (13.6)	83.8 (14.2)	82.6 (13.0)	0.179
MetS prevalence [%] *	53 [6.0]	14 [3.0]	39 [9.0]	<0.001
Number of components n [%] *				
0	355 [39.9]	236 [51.0]	119 [27.9]	<0.001
1	331 [37.2]	148 [32.0]	183 [42.9]	
2	151 [17.0]	65 [14.0]	86 [20.1]	
3	39 [4.4]	11 [2.4]	28 [6.6]	
4 or more	14 [1.6]	3 [0.6]	11 [2.4]	
Tobacco (≥10 cigarettes per week) [%] *	60 [6.7]	22 [4.7]	30 [7.2]	0.349
Alcohol (≥1 times per week) [%] *	92 [10.1]	42 [9.0]	50 [11.7]	0.041
PA levels (≥150 min per week) [%] *	293 [33.0]	146 [31.6]	150 [35.3]	<0.001

Significant between-sex differences for Student’s *t*-test or χ^2^*; ^a^ Mediterranean diet quality: (1) ≤3 points = poor diet quality; (2) 4–7 points = average diet quality; and (3) ≥8 points = good diet quality (optimal Mediterranean diet style). BMI, body mass index; MetS, metabolic syndrome; PA, physical activity.

**Table 2:** Anthropometric, body composition, cardiovascular risk factors, and associated factors by metabolic syndrome status.

Characteristics	Metabolic Syndrome Status		
	No	Yes	*p* Value
Anthropometric and body composition			
Age (years)	21.4 (3.2)	23.1 (4.0)	<0.001
Weight (kg)	58.7 (9.2)	82.7 (15.4)	<0.001
Height (cm)	163.7 (8.6)	170.5 (9.1)	<0.001
Body mass index (kg/m^2^)	21.8 (2.6)	28.4 (4.3)	<0.001
Waist circumference (cm)	71.1 (6.5)	90.8 (11.1)	<0.001
Waist to height ratio	0.435 (0.038)	0.534 (0.063)	<0.001
Body fat (%)	20.8 (7.6)	27.9 (8.4)	<0.001
Lean mass (kg)	59.4 (10.4)	45.3 (7.4)	<0.001
Cardiovascular risk factors			
Systolic blood pressure (mmHg)	112.7 (9.4)	130.0 (13.0)	<0.001
Diastolic blood pressure (mmHg)	70.8 (6.7)	84.5 (14.2)	<0.001
Mean blood pressure (mmHg)	84.7 (6.7)	99.7 (11.1)	<0.001
Total cholesterol (mg/dL)	133.0 (29.7)	154.1 (37.7)	<0.001
Triglycerides (mg/dL)	79.0 (25.2)	180.3 (75.4)	<0.001
LDL-c (mg/dL)	85.7 (30.4)	87.4 (29.3)	<0.001
HDL-c (mg/dL)	52.3 (9.9)	31.7 (7.5)	0.125
Glucose fasting (mg/dL)	79.8 (12.0)	92.1 (16.1)	<0.001
Associated factors *			
Women	14 [26.4]	449 [53.6]	<0.001
Men	39 [73.6]	388 [46.4]	
Age group *			
18 to 19 years	12 [22.6]	306 [36.6]	<0.001
20 to 22 years	15 [28.3]	313 [37.4]	
Over 23 years	26 [49.1]	218 [26.0]	
Level of adiposity (%) *			
<20	8 [15.1]	405 [48.4]	0.002
20 to 29	27 [50.9]	291 [34.8]	
30 to 39	14 [26.4]	125 [14.9]	
>40	4 [7.5]	16 [1.9]	
Nutritional status *			
Underweight	0 [0.0]	29 [3.5]	<0.001
Normal	6 [11.3]	609 [72.8]	
Overweight	31 [58.5]	166 [19.8]	
Obesity	16 [30.2]	33 [3.95	
Mediterranean diet quality *			
Low	15 [28.3]	418 [49.9]	0.081
Medium	34 [64.2]	346 [41.3]	
High	4 [7.5]	73 [8.7]	
Ethnic group *			
Indigenous	8 [15.1]	16 [1.9]	0.210
Afro-Colombian	36 [67.9]	208 [24.9]	
Mestizo	9 [17.0]	613 [73.2]	

Data are shown as (mean (SD) or [frequencies]). Significant between-sex differences for Student’s *t*-test or χ^2^*.

## Abbreviations

The following abbreviations are used in this manuscript:

BF%	body fat percentage
BIA	Bioelectrical impedance analysis
BMI	body mass index
DBP	diastolic blood pressure
ENSIN, 2010	Colombian national representative nutrition survey
FUPRECOL study	Association between Muscular Strength and Metabolic Risk Factors in Colombia
HDL-c	low high-density lipoprotein cholesterol
IDF	International Diabetes Federation
LDL-c	low-density lipoprotein cholesterol
MetS	metabolic syndrome
NHANES	National Health and Nutrition Examination Survey
SBP	systolic blood pressure
WC	waist circumference
WHtR	waist-to-height ratio
